# The study on the function and cell source of interleukin‐6 in interstitial cystitis/bladder painful syndrome rat model

**DOI:** 10.1002/iid3.505

**Published:** 2021-08-18

**Authors:** Zhenming Zheng, Jiapeng Zhang, Caixia Zhang, Wenshuang Li, Kaiqun Ma, Hao Huang, Kuiqing Li, Yousheng Yao

**Affiliations:** ^1^ Guangdong Provincial Key Laboratory of Malignant Tumor Epigenetics and Gene Regulation, Sun Yat‐sen Memorial Hospital Sun Yat‐sen University Guangzhou China; ^2^ Department of Urology, Sun Yat‐sen Memorial Hospital Sun Yat‐sen University Guangzhou China; ^3^ Guangdong Provincial Clinical Research Center for Urological Diseases Guangdong China; ^4^ Department of Urology West China Hospital of Sichuan University Sichuan China

**Keywords:** cell source, interleukin‐6, interstitial cystitis/bladder painful syndrome, macrophages

## Abstract

**Objective:**

The elevated expression of interleukin‐6 (IL‐6) in patients with interstitial cystitis/bladder painful syndrome (IC/BPS) has been demonstrated, but the role of IL‐6 in IC/BPS and its source remain to be explored.

**Methods:**

IC/BPS rat model was created in female rats by using long‐term intermittent intravesical hyaluronidase (0.5 ml, 4 mg/ml). After modeling, IL‐6 stimulation group, and anti‐IL‐6R group were treated with recombinant rat IL‐6 and tocilizumab, respectively. Symptomatic changes were detected by Vonfrey pain score and urodynamics, and hematoxylin‐eosin (HE) staining, mast cell staining and Masson staining were used to evaluate the changes of inflammation in the bladder tissue of rats. Cell sources of IL‐6 was explored through enzyme linked immunosorbent assay (ELISA) test, reverse transcription polymerase chain reaction (RT‐PCR), and western‐blot test on the supernatant of coculturing rat bladder epithelial cells and rat macrophages.

**Results:**

The Vonfrey pain scores of the model group and IL‐6 stimulation group were significantly higher than those of the control group, while the anti‐IL‐6R group were significantly lower (*p *< .05). Compared with the blank control group, urodynamic results showed that the urination interval of the model group and IL‐6 stimulation group was significantly shortened, and the maximum bladder capacity was significantly reduced (*p* < .05), and anti‐IL‐6R treatment significantly alleviated the inflammatory response of bladder tissue. The results of HE, Mast cell staining, and Masson staining showed that the inflammatory response of bladder tissue after anti‐IL‐6R treatment was significantly reduced. Through cells coculture, the relative expression of IL‐6 from model group was found significantly higher than blank control group by RT‐PCR, ELISA, and western blot test (*p* < .05).

**Conclusions:**

IL‐6 played an essential role in the development of IC/BPS rat model as a proinflammation cytokine. Further evidence from coculture proved that macrophages are the cell resource of IL‐6 in IC/BPS.

## Graphical abstract

This study found that interleukin‐6 plays an important role as a proinflammatory factor in the inflammatory progression of interstitial cystitis/bladder painful syndrome (IC/BPS), which is mainly derived from the synthesis and secretion of macrophages.

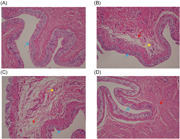

## 1 INTRODUCTION

Interstitial cystitis/bladder pain syndrome (IC/BPS) is a clinical diagnosis based on frequent urination, urgency, and symptoms of bladder and/or pelvic pain.^1^ IC/BPS patients with frequent urination, urgent urination, bladder pain symptoms seriously affect their work and life, they often suffer from sleep disorders, depression, anxiety, stress, social dysfunction, and sexual dysfunction.^2^ However, at present, the pathogenesis of IC/BPS has not been fully clarified in the world, and there is no unified diagnostic standard and treatment method, and the treatment effect is not ideal.^3^, ^4^ Therefore, it is urgent to carry out more in‐depth research on its pathogenesis and treatment.

Among the numerous etiological theories about IC/BPS, the immune correlation theory has been recognized and concerned by the majority of peer experts.^5^, ^6^ Based on the theory that IC/BPS is an autoimmune disease, anti‐TNF α agents such as adalimumab, certolizumab pegol, and other biological agents have been proved to significantly improve the symptoms of IC/BPS through clinical trials.^7^, ^8^ In addition, there were a large number of immune‐related studies on IC/BPS have also found that the inflammatory progression of IC/BPS is related to the expression levels of inflammatory cytokines such as interleukin 6 (IL‐6), IL‐17, and IL‐1β, and is also related to mast cell activation.^9^, ^10^, ^11^ In our previous study, we demonstrated that the expression level of IL‐6 was positively correlated with IC/BPS inflammation,^12^ which may become a new target for the etiological research and treatment of IC/BPS.

Macrophage is a kind of immune cells with a variety of functions. In the inflammatory response, macrophage has the function of presenting antigen, phagocytosis, participating in the immune regulatory response, and can also produce a variety of cytokines and inflammatory factors to regulate the inflammatory response.^13^ Monocytes and macrophages activated by toll‐like receptors were the primary sources of IL‐6 synthesis in acute inflammation.^14^ However, in chronic inflammatory of IC/BPS, there was no relevant study on the source and upstream regulatory pathway of IL‐6 which is also highly expressed. This study aims to further explore the role and cell sources of IL‐6 in IC/BPS inflammatory response based on the previous research results of our research group.

## 2 METHODS

### 2.1 Construction and treatment of IC/BPS rat model

Sixty adult female Sprague‐Dawley rats (weight 180–220 g) were purchased and kept in the Experimental Animal Center of Sun Yat‐sen University, and all experimental protocols and procedures were approved by the Sun Yet‐sen University Institutional Animal Care and Use Committee. Sixty rats were randomly divided into four groups (Blank control group, model group, IL‐6 stimulation group, IL‐6R antagonist group) with 15 rats in each group, all rats were fed a standard pallet laboratory chow and allowed access to tap water freely. Rats were anesthetized under pentobarbital sodium (40 mg/kg) though transperitoneal injection. In model group, IL‐6 stimulation group and IL‐6R antagonist group, a PE‐50 catheter was inserted into the bladder through the urethra to empty the bladder, and 0.5 ml hyaluronidase solution (0.4 ml, 4 mg/ml) was infused intravesical and remained for 30 min. In blank control group, 0.4 ml saline was infused respectively following the same procedure. After all rats got treated every 3 days for a month, the IC/BPS rat model was established, then VonFrey pain score and urodynamics were used to initially verify the model's simulacra. Next, we gave rats in the IL‐6 stimulation group intermittent subcutaneous injections of exogenous IL‐6 solution (1,000,000 U, in 2 ml saline), once every 3 days, 10 times in total, and the IL‐6R antagonist group received intravenous infusion of IL‐6R Antagonist (tocilizumab 4 mg/ml, adding 2 ml physiological saline solution, single intravenous infusion for 1 h), once a week, four times in total.

### 2.2 VonFrey brush test

After the model group was established and the treatment of each treatment group was completed. We put these rats on a stimulation platform whose four walls and top wall are covered with glass, and the bottom wall is a mesh structure. After these rats adapt to the new platform environment, the lower abdomen and perineum area of the rats are stimulated with 0.5, 1, and 5 g stimulus from the inferior wall (5–10 s/time, 20–30 s interval). Repeat 10 times and record the total score (0 points: no significant response; 1 point, torso posture change; 2 points, jumped when stimulated).

### 2.3 Urodynamic test

Before urodynamic test, rats were prohibited from drinking water for 12 h. After intraperitoneal injection anesthesia, a PE 50 ureteral catheter was inserted into the bladder of rats and filled at a rate of 10 ml/h. The ureteral catheter was connected to the syringe pump and pressure sensor through a three‐way cock. The urination interval, urination volume, maximum bladder pressure, and corresponding maximum bladder capacity were recorded.

### 2.4 Bladder inflammation degree evaluation

#### 2.4.1 Hematoxylin‐eosin (HE) staining

After the rats were killed by carbon dioxide asphyxiation, we obtained the rat's bladder tissue. Bladder section was divided and fixed in 10% formalin for further paraffin fixed slice. With the help of pathologists from the department of pathology, Sun Yat‐sen Memorial hospital, fixed bladder tissue was embedded in paraffin, cut into 5‐mm transverse sections, and stained with HE later on. Inflammation degree of bladder tissue from different groups was assessed through observation on the number of microvascular vessels in the bladder, the degree of interstitial fibrosis and the proliferation of epithelial cells.

#### 2.4.2 Masson staining

For better evaluate the effect of IL‐6 and IL‐6R antagonist treatment on interstitial fibrosis, bladder tissue slices were stained by Masson staining kit (Solarbio) according to the manufacturer's instructions.

#### 2.4.3 Mast cells staining

The activation of mast cells is believed to be closely related to the inflammatory response of IC/BPS. To evaluate the degree of inflammation, we performed toluidine blue staining on rat bladder tissues in each group and counted the number of mast cells. We strictly follow the instructions of the kit manufacturer.

### 2.5 Coculture system

#### 2.5.1 Primary culture of bladder epithelial cells in vitro

After the rats from blank control group and model group were killed, the bladder epithelial tissue with a size of 3 mm × 3 mm was cut with ophthalmic scissors and then loaded into a 5 ml centrifuge tube containing D‐Hank's solution, then transferred to the laboratory quickly. In sterile workbench, squeezed out the blood in the tissue, the mucosal layer on the surface of the bladder tissue was carefully separated by ophthalmic scissors then discarded the remaining tissue, and the mucosal tissue was rinsed again with D‐Hank's solution for 3–5 times and transferred to a new culture dish. The mucosa layer was cut into 1 mm × 1 mm size then transferred to a culture flask. Evenly placed the tissues on the bottom of the culture bottle. Add serum‐free keratin medium (KSFM, containing 5 ug/L rat recombinant epithelial cytokine and 50 ug/L bovine pituitary extract) and cultured in 37°C incubator for 4 h.

#### 2.5.2 Isolation and culture of rat peritoneal macrophages

SD rats in 2–3 months age were sacrificed by cervical dislocation. After soaking for 7 min with 75% alcohol, the rats were lifted upside down and injected intraperitoneally into the lower abdomen with 10 ml of serum‐free Dulbecco's modified Eagles medium (DMEM). The abdomen was gently rubbed for 2–3 min, then allowed to stand for 6 min, and the abdominal cavity of the rat was opened in a sterile environment. After observing and confirming that the intestinal tube was flattened and a pale‐yellow peritoneal fluid was observed, 5–10 ml of peritoneal fluid was withdrawn by a syringe. After low‐speed centrifugation, the supernatant was discarded, and the cell concentration was adjusted in a conventional DMEM medium (containing 10% fetal calf serum, 1% cyan‐streptomycin antibody), and then inoculated into a new culture flask, and cultured at 37°C in a cell culture incubator.

#### 2.5.3 Coculture

Digest the 3rd generation of primary bladder epithelial cells (seeding in lower chambers with cell density: 5 × 10^5^/cm^2^) and cocultured with rat macrophages (seeding in upper chambers with cell density: 4 × 10^5^/cm^2^) in transwell plate. 24 h after plating, the supernatant of the macrophage culture supernatant was collected for subsequent tests.

### 2.6 Analysis of IL‐6 expression

Reverse transcription polymerase chain reaction (RT‐PCR) was performed in 25ul volume system using a SYBR Green PCR Master Mix. Primer sequences were as follows: IL‐6, sense, AGACTTCACAGAGGATACCACCCAC, antisense, CAATCAGAATTGCCATTGCACAA; GAPDH, sense, CAACGGGAAACCCATCACCA, antisense, ACGCCAGTAGACTCCACGACAT. Antibodies for Western‐blot test include Anti‐IL‐6 (ab9324, ABCAM) and Anti‐GAPDH (ab181602; ABCAM). The levels of IL‐6 in macrophage culture supernatant was assessed by rat IL‐6 ELISA kit (Multi Science).

## 3 RESULTS

### 3.1 Changes in biological behavior

**Figure 1:**
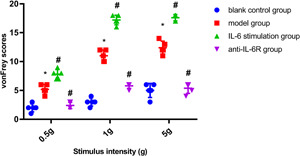
vonFrey pain score of blank control group, model group, IL‐6 stimulation group, and anti‐IL‐6R group. The vonFrey scores of the rats in the model group and IL‐6 stimulation group were significantly higher than those in the blank control group, whereas they were decreased significantly after IL‐6R antagonistic treatment, but still higher than the blank control group. *p < .01 versus the blank control group, ^#^ *p* < .01 vs the model group. IL, interleukin

The vonFrey brush test scores of the rats in the model group and IL‐6 stimulation group were significantly higher than those in the blank control group (*p* < .05) (Figure 1). In contrast, the scores were significantly decreased after IL‐6R antagonistic treatment, but still higher than the blank control group (*p* < .05) (Figure 1). Compared with the blank control group, the urination interval of the model group and IL‐6 stimulation group was significantly shortened, and the maximum bladder capacity was significantly reduced when the bladder pressure was maximum, while rats under IL‐6R antagonist treatment showed prolonged urination interval and partial recovery of bladder maximum capacity (*p* < .01, Table 1).

**Table 1:** Comparison of urodynamic parameters in each group

Groups	Urination interval (s)	Maximum bladder capacity (ml)	Maximum bladder pressure (mmHg)
Blank control	486 ± 33	1.31 ± 0.26	27.32 ± 2.21
Model	185 ± 16^#^	0.43 ± 0.04^#^	26.04 ± 1.87
IL‐6 stimulation	115 ± 19* ^,^ ^#^	0.46 ± 0.05^#^	25.56 ± 1.94
IL‐6R antagonist	337 ± 45*	1.16 ± 0.12*	29.10 ± 2.04

*Note:* Compared with the blank control group, the urination interval of the model group and IL‐6 stimulation group was significantly shortened, and the maximum bladder capacity was significantly reduced when the bladder pressure was maximum, while rats under IL‐6R antagonist treatment showed prolonged urination interval and partial recovery of bladder maximum capacity.

Abbreviation: IL, interleukin.

* *p* < .01 versus the model group.

^#^ *p* < .01 versus the blank control group.

### 3.2 Inflammatory changes of rat bladder tissue after IL‐6 and IL‐6R treatment

**Figure 2:**
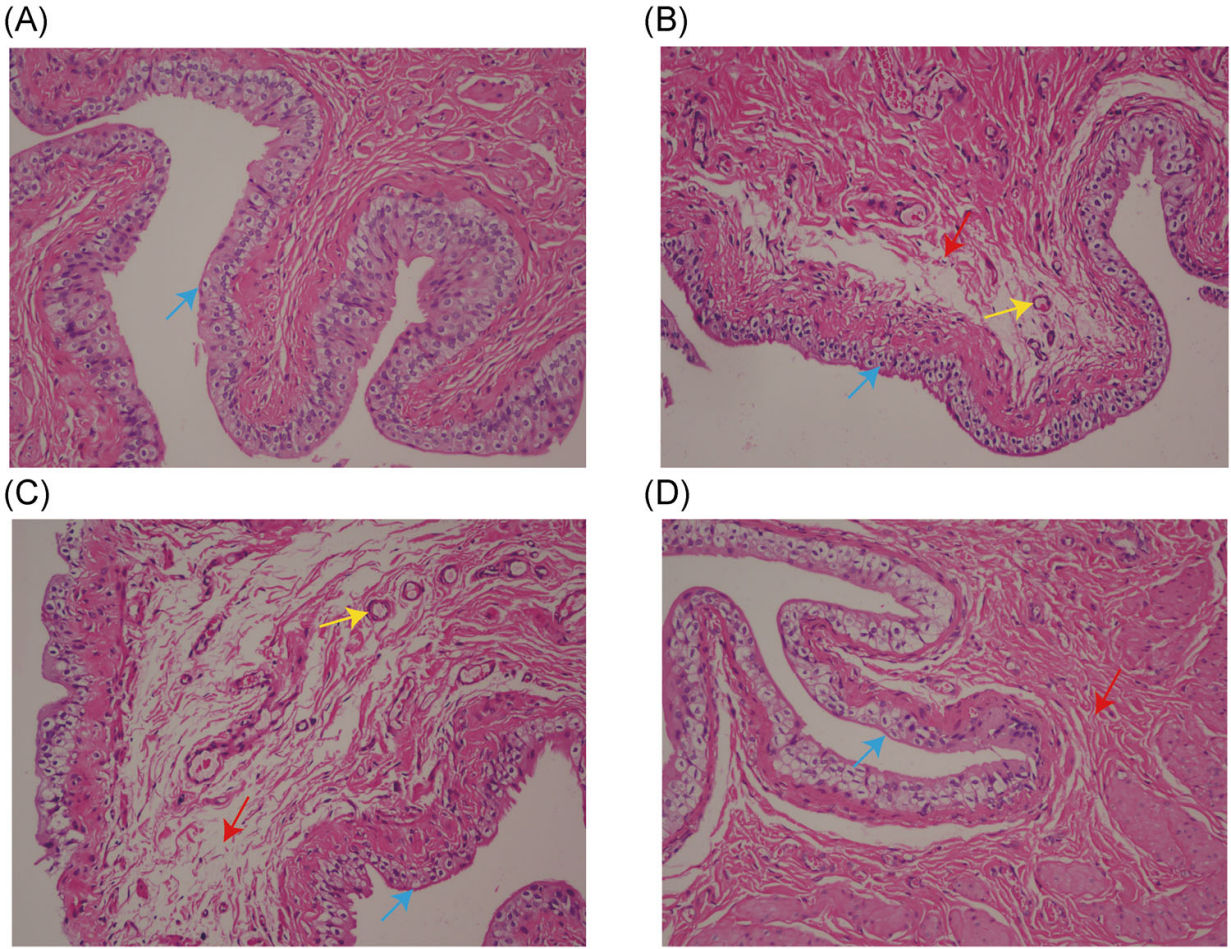
HE staining of bladder tissue from all groups. (A) blank control group, (B) model group, (C) IL‐6 stimulation group, and (D) IL‐6R antagonist group (magnification times: ×200, blue arrow points for bladder epithelia cells, yellow arrow points interstitial vessels; red arrow points interstitial collagenous fiber). The inflammation degree of the model group and IL‐6 stimulation group was significantly higher than that of the blank control group and IL‐6R antagonist treatment group, manifested by thinning of the bladder epithelium, increased number of interstitial micro vessels, and proliferation of collagen fibers. HE, hematoxylin‐eosin; IL, interleukin

**Figure 3:**
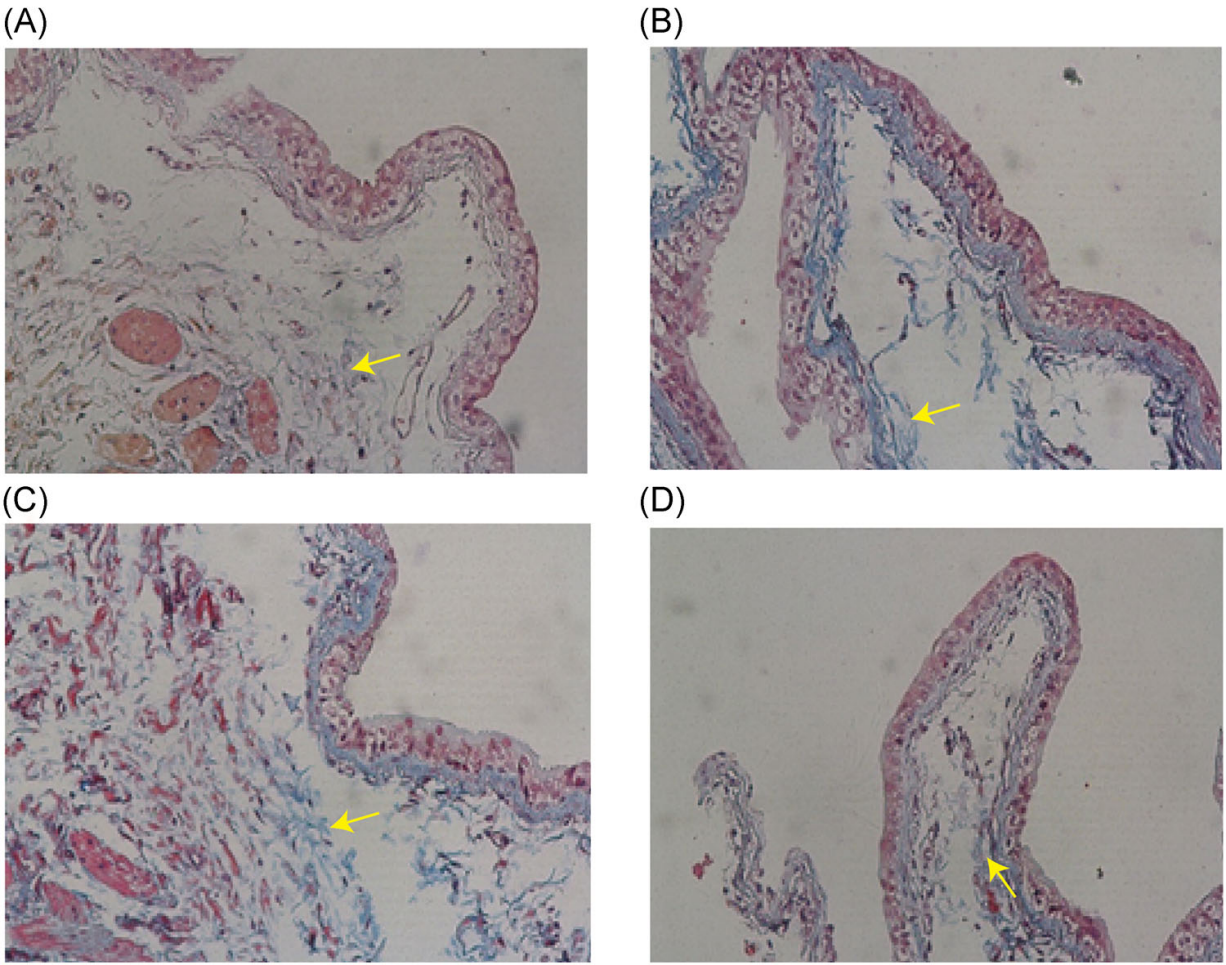
Masson staining of bladder tissue from all groups. (A) blank control group, (B) model group, (C) IL‐6 stimulation group, (D) IL‐6R antagonist group (magnification times: ×100, yellow arrows point for blue stain of collagenous fiber). The degree of interstitial fibrosis in the IL‐6 stimulation group was significantly higher than that in the model group, while IL‐6R antagonist treatment was significantly reduced. IL, interleukin

**Figure 4:**
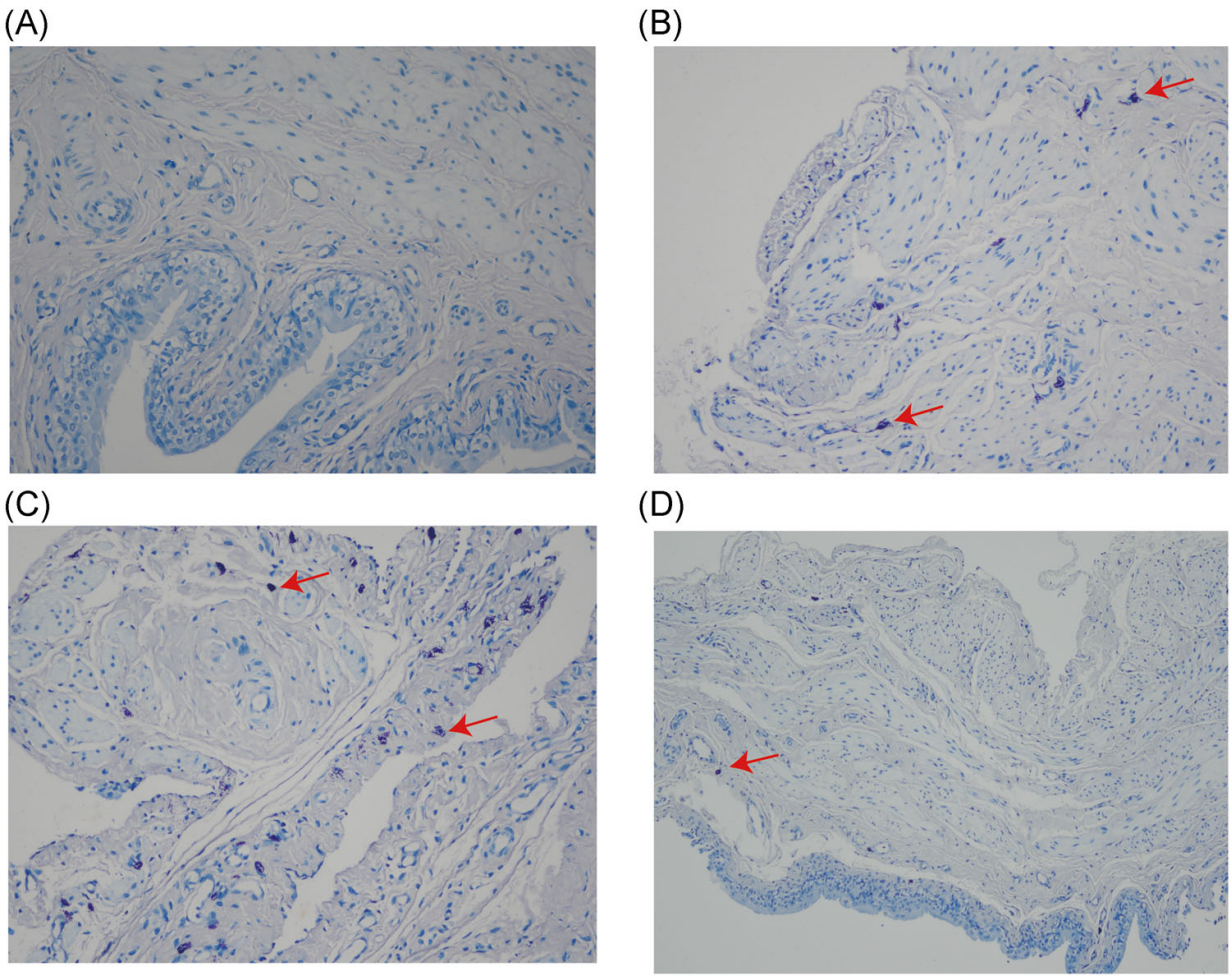
Toluidine blue staining staining of bladder tissue from all groups. (A) blank control group, (B) model group, (C) IL‐6 stimulation group, (D) IL‐6R antagonist group (magnification times: ×200, red arrows point for mast cells). The number of mast cells in the IL‐6 stimulation group was significantly higher than that in the model group, while the IL‐6R antagonist treatment group was significantly reduced (*p* < .05). IL, interleukin

HE staining (Figure 2) showed that the inflammation degree of the model group and IL‐6 stimulation group was significantly higher than that of the blank control group and IL‐6R antagonist treatment group, manifested by thinning of the bladder epithelium, increased number of interstitial micro vessels, and proliferation of collagen fibers. Masson staining (Figure 3) showed that the degree of interstitial fibrosis in the IL‐6 stimulation group was significantly higher than that in the model group, while IL‐6R antagonist treatment was significantly reduced. Mast cell staining (Figure 4) results showed that the number of mast cells in the IL‐6 stimulation group was significantly higher than that in the model group, while the IL‐6R antagonist treatment group was significantly reduced (*p* < .05). These results all proved that IL‐6 can aggravate the inflammatory response of IC/BPS, and IL‐6 antagonists can reduce the inflammatory response of IC/BPS.

### 3.3 Changes of IL‐6 expression level in coculture system

**Figure 5:**
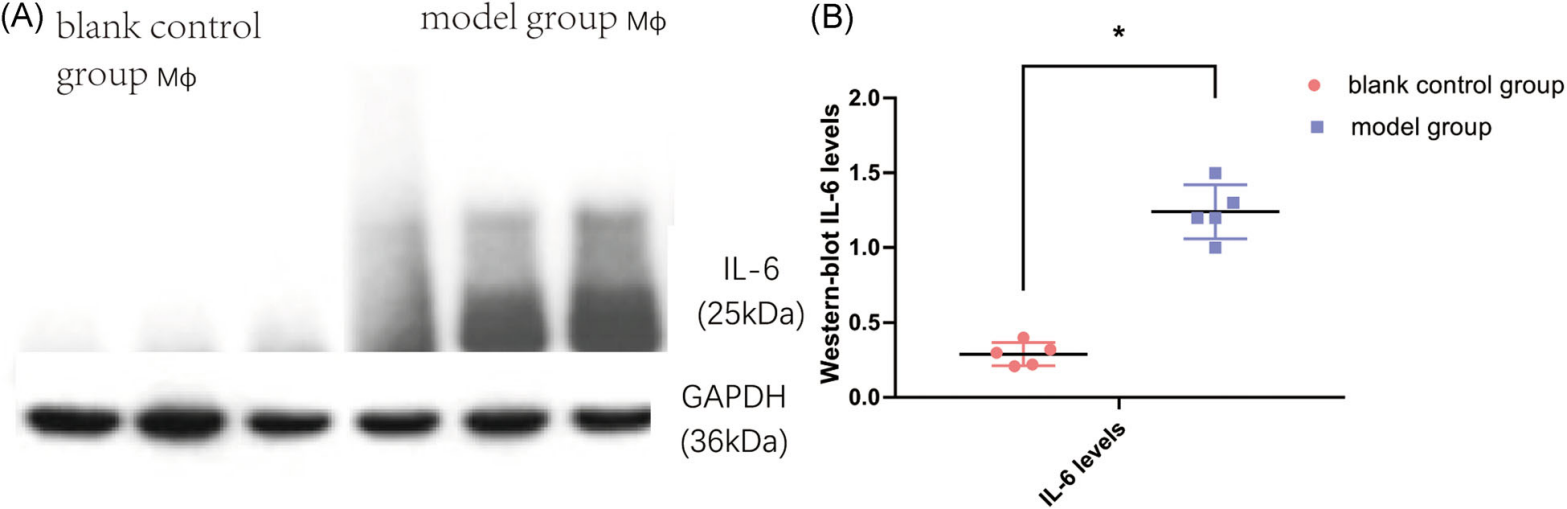
Western‐blot test of IL‐6 levels in different groups of cocultured macrophages. IL‐6 levels were normalized by respective bladder weight. IL‐6 levels were significantly elevated in macrophages cocultured with the model group (*p < .05). IL, interleukin

**Figure 6:**
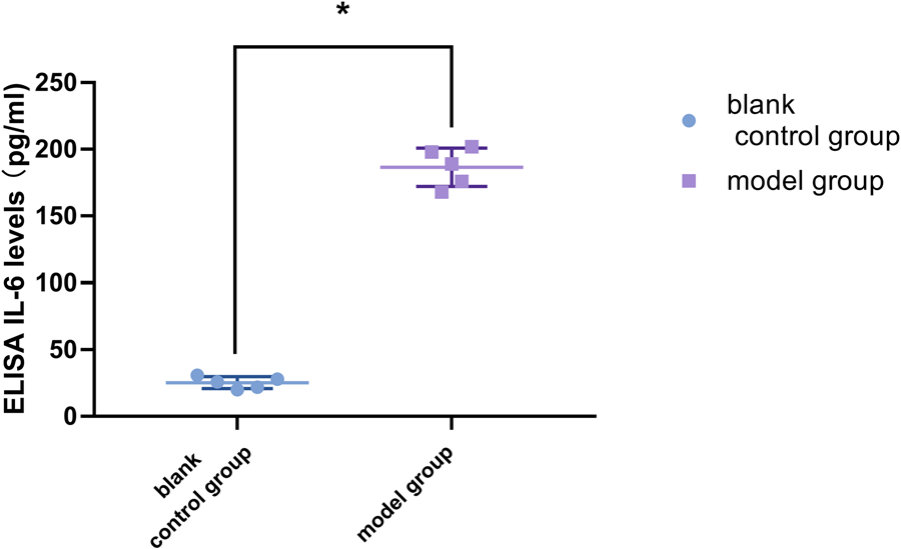
ELISA of IL‐6 levels in supernatant fluid of two groups of cocultured macrophages. IL‐6 levels were normalized by respective bladder weight. IL‐6 levels were significantly enhanced in the supernatant of macrophages cocultured with model group bladder epithelial cell compared to that of blank control group (*p < .05). Values are mean ± SD. ELISA, enzyme linked immunosorbent assay; IL, interleukin

**Figure 7:**
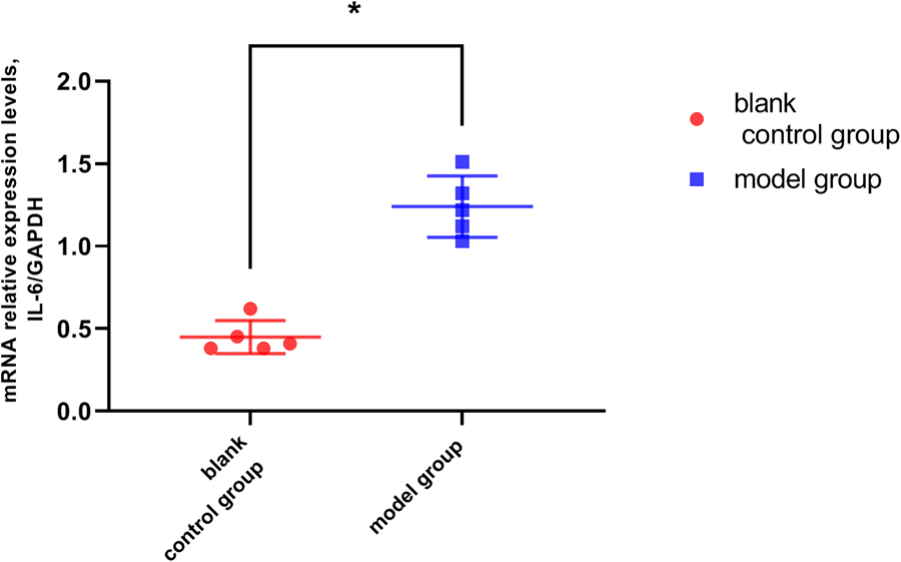
RT‐PCR of IL‐6 mRNA in different groups of cocultured macrophages. Relative quantities of IL‐6 mRNA were normalized by GAPDH mRNA. Relative IL‐6 mRNA expression was significantly enhanced in the supernatant of macrophages cocultured with model group bladder epithelial cell compared to that of blank control group (*p < .05). GAPDH, glyceraldehyde 3‐phosphate dehydrogenase; IL, interleukin; mRNA, messenger RNA; RT‐PCR, reverse transcription polymerase chain reaction

Western‐blot test results showed that the expression levels of IL‐6 in the supernatant of macrophages cocultured with the model group was significantly higher than that in the blank control group (*p* < .05, Figures 5A, 5B), and enzyme linked immunosorbent assay (ELISA) tests also showed similar results (*p* < .05, Figure 6). The RT‐PCR results also showed that the relative expression levels of interleukin‐6 RNA in macrophages cocultured with epithelial cells in the model group was much higher than that in the blank control group (*p* < .05, Figure 7). These results suggested that macrophages have the function of synthesizing and secreting IL‐6 in IC/BPS inflammatory microenvironment, and that macrophages may be the main cell source of high expression of IL‐6 in IC/BPS model rat bladder tissue.

## 4 DISCUSSION

The pathogenesis of IC/BPS is currently unclear, and a variety of etiological theories have been proposed. The more promising ones include: epithelial permeability theory, mast cell activation theory, and immune neuroinflammation theory.^4^, ^10^ The three theories are not contradictory and closely related. Combined with the findings of previous studies, we speculated that the pathogenesis of IC/BPS may be a comprehensive factor: the protective GAG layer on the bladder mucosal surface decreases, and the permeability of the bladder epithelium increases; after potassium ion‐based chemicals infiltrate the bladder interstitial through osmosis, they cause contact injury and inflammation, and stimulate painful nerves, leading to pain‐based clinical symptoms. The inflammatory response further stimulates mast cells, macrophages, and so forth, to produce and release IL‐6 and other inflammatory mediators, which makes the progress of inflammation aggravate and eventually forms chronic neuroinflammation. Clinically, the symptoms of IC/BPS patients are often progressively worsening. This phenomenon may be closely related to the vicious circle triggered by cellular immunity in the inflammatory process.

Although there are many methods to construct IC/BPS disease models at present, they all had shortcomings in different aspects^15^, ^16^: (1) most models can only simulate part of the disease; (2) The defects of the existing animal models based on the principle of bladder epithelial permeability lie in excessive or insufficient damage of the bladder; (3) The identification of the model was mainly based on pathological indicators, and the pain detection indicators were lacking. (4) Most of them were acute bladder inflammation models, but lack of pseudo‐chronic bladder inflammation models. Therefore, according to the theory of epithelial permeability, which is one of the initiators of IC/BPS, we established a rat model of IC/BPS by intermittent continuous bladder perfusion with hyaluronidase. Compared with the direct destruction of bladder mucosal epithelium by chemical modeling, hyaluronidase has the advantage of selectively destroying the GAG layer, thus achieving the purpose of constructing chronic inflammation model. It has been verified that compared with the existing IC/BPS animal model, our original IC/BPS rat model has better analog authenticity in terms of both inflammation‐related molecular changes and symptomatic changes such as frequency of urine and perineal pain.^17^

As a multieffect cytokine, IL‐6 has been confirmed by many studies in its pathogenic effects in inflammatory diseases.^18^ Lotz et al.^19^ reported for the first time that IL‐6 expression increased in the urine of IC/BPS patients. Our previous research results further confirmed that the expression level of IL‐6 was positively correlated with the symptoms of IC/BPS patients.^12^ In addition, there some studies have found that IL‐6 has the effect of enhancing the detrusor contraction function, which may be the cause of the increased urination frequency in IC/BPS patients.^20^, ^21^ However, there is no relevant research on the specific effect and mechanism of IL‐6 on IC/BPS.

By treating rats with exogenous IL‐6 stimulation and IL‐6R antagonist, we confirmed that exogenous IL‐6 stimulation can aggravate the pain symptoms in the lower abdomen and perineal area, shorten the urination interval and reduce the maximum bladder capacity in model rats. Bladder epithelial cell proliferation, interstitial fibrosis and microvascular proliferation are aggravated in bladder tissue. In the contrast, intervention with IL‐6R antagonist (tocilizumab) succeed to repair IC/BPS inflammation induced by hyaluronidase perfusion, which is manifested by the relief of urinary frequency symptoms, recovery of bladder capacity and reduction of the degree of interstitial fibrosis and reduction of epithelial cell proliferation. Based on a more realistic IC/BPS rat model, this study elucidated the promoting effect of IL‐6 on the inflammatory progression of IC/BPS, and confirmed that IL‐6 is one of the key factors in the role of inflammation, which provided a theoretical and experimental basis for the further study of IL‐6 in the etiological mechanism of IC/BPS.

After primary cultured of rat bladder epithelial cells in each group for three generations, they were cocultured with macrophages derived from the abdominal cavity. The IL‐6 expression levels in the supernatant were detected by western‐blot, RT‐PCR, and ELISA, and the results showed that the IL‐6 expression levels in the supernatant of macrophages cocultured with bladder epithelial cells in the blank control group were significantly lower than that in the supernatant of macrophages cocultured with the model group. These results showed that macrophages may be the main cell source of IL‐6 highly expressed in the bladder tissue of IC/BPS model rats. In addition, IL‐6 with the characteristics of multi‐cell origin may also be partly derived from the proliferating fibroblasts and endothelial cells in the interstitial of the bladder tissue, which we will further explore in the next step. In the future, we will further explore the upstream pathway of regulating the synthesis and secretion of IL‐6 by macrophages, and whether macrophages can participate in the progression of IC/BPS by regulating the direction and proportion of their own polarization and whether they can synthesize and secrete other cytokines. The exploration of these regulatory mechanisms and inflammatory pathways is expected to further clarify the pathogenesis of IC/BPS and provide new ideas for the treatment of IC/BPS.

Admittedly, there were still some shortcomings in this study: first, this study has proved that IL‐6 has a pro‐inflammatory effect in IC/BPS model rats, and IL‐6R has the effect of reducing inflammation, while without further exploring the upstream pathway regulating the synthesis and secretion of IL‐6 by macrophages. Second, there were many sources of IL‐6, in this study, only macrophages were identified as the main cell sources of IL‐6, and whether bladder epithelial cells and fibroblasts were also involved in the synthesis and secretion of IL‐6 was not explored. These were also the directions for our next study.

## 5 CONCLUSION

In this study, we investigated the influence of IL‐6 on the occurrence and progression of IC/BPS in the rat model of IC/BPS, and further explored the cell source of highly expressed IL‐6, and drew the following conclusions: IL‐6 may be a key inflammatory mediator in the occurrence and progression of IC/BPS, and has a direct influence on the development of inflammation in animal models. In the IC/BPS inflammatory microenvironment, the synthesis and secretion of macrophages are one of the main sources of the high expression of IL‐6.

## CONFLICT OF INTERESTS

The authors declare that there are no conflict of interests.

## AUTHOR CONTRIBUTIONS

Yousheng Yao designed the study and took overall control of the manuscript. Zhenming Zheng and Jiapeng Zhang drafted the manuscript. Zhenming Zheng, Jiapeng Zhang, Wenshuang Li, and Kaiqun Ma were involved with experimental and analytical aspects of the manuscript. Hao Hang and Kuiqing Li helped with study design and data analysis. Yousheng Yao and Caixia Zhang reviewed and helped the revision of the manuscript. All authors agree to be accountable for the content of the work.

## Data Availability

Datas could be obtained upon request to the corresponding author.
